# Psychosocial Burden of Adolescent Idiopathic Scoliosis: A Systematic Review and Meta‐Synthesis of Patient and Caregiver Perspectives

**DOI:** 10.1111/hex.70785

**Published:** 2026-08-06

**Authors:** Shuang Wang, Jiayi Zhao, Xiong Hu, Yanli Yuan, Yingying Xu, Ting Kan, Lingyan Cheng, Chen Qiu

**Affiliations:** ^1^ Department of Orthopedics Fourth Medical Center Chinese PLA General Hospital Beijing China; ^2^ Department of Surgery The Second Naval Hospital of Southern Theater Command of PLA Sanya Hainan China; ^3^ Graduate School Chinese PLA General Hospital Beijing China

**Keywords:** adolescent idiopathic scoliosis, caregivers, meta‐synthesis, psychological aspects, qualitative research

## Abstract

**Background:**

Adolescent idiopathic scoliosis (AIS) is a three‐dimensional spinal deformity that occurs in adolescents, exerting multidimensional and bidirectional impacts on the physical and mental health and social functioning of both patients and their caregivers. Prevalent misconceptions and stigma, insufficient psychological care in clinical practice, and inadequate social support may further exacerbate their negative psychosocial experiences.

**Objective:**

This review aimed to integrate and elucidate the core themes and evolving mechanisms of the psychosocial experiences of adolescents with AIS and their caregivers, reveal the interactional dynamics between the two groups throughout the disease trajectory, and provide an evidence‐based foundation for optimising healthcare and social support.

**Methods:**

Based on the qualitative evidence synthesis (QES) framework, the meta‐aggregation method developed by the Joanna Briggs Institute (JBI) was employed. A systematic search of PubMed, Embase, CINAHL, PsycINFO, and Web of Science was conducted from database inception to April 2026. Qualitative studies exploring the psychological burden and lived experiences of adolescents with AIS and their caregivers were included.

**Results:**

Nineteen qualitative studies from 10 countries, involving 323 participants, were included. At the patient level, three core themes were synthesized: acute shock and multidimensional responses during the initial diagnosis phase; dynamic adaptation and transient positive experiences during the treatment phase; and divergent pathways of posttraumatic growth and residual trauma during the recovery phase. At the caregiver level, three core themes were identified: a structural burden comprising both resource safeguarding and logistical demands; an imbalance in support systems characterized by a deficiency in professional guidance and disruption of family equilibrium; and a progression of emotional exhaustion from self‐blame and intense pressure to depressive symptomatology. Furthermore, a dynamic of conflictual intimacy, encompassing both direct interactive conflict and enmeshment within the family system, emerged between patients and caregivers.

**Conclusion:**

The psychological experiences of adolescents with AIS evolve through distinct temporal phases, while caregivers' psychological burden follows a cascading pathway from structural strain to emotional exhaustion. The two parties are locked in a bidirectional interaction shaped by the structural tension between parental protection and adolescent autonomy. Healthcare professionals should tailor psychological support to the patient's disease stage, ensure transparent information delivery, and provide transitional training for caregivers to mitigate the transmission of stress. At the societal level, it is essential to strengthen early screening and public education, eliminate stigma, and provide accessible support to help AIS families navigate role transitions throughout the disease course.

**Patient Contribution:**

Two adolescents with AIS and two family caregivers participated as co‐investigators in this review. They contributed to refining the research question, prioritizing outcomes of importance, interpreting the findings, and reviewing the manuscript for clarity and relevance.

## 1 Background

Adolescent idiopathic scoliosis (AIS) is defined as a three‐dimensional spinal deformity with a Cobb angle ≥ 10° in the absence of identifiable causes, such as congenital malformations or neuromuscular disorders [1]. AIS primarily occurs in adolescents aged 10 and 18 years of age and represents the most common type of scoliosis, accounting for approximately 80% of all cases [2]; its global prevalence ranges from 0.47% to 5.20% [3], with a higher prevalence among females [4]. Rapid growth during puberty increases the risk of ongoing curve progression and precipitates a cascade of physiological burdens: three‐dimensional deformities of the spine and thorax lead to postural abnormalities and body asymmetry; malalignment and myofascial compensation are associated with chronic or activity‐related pain; reduced flexibility and endurance restrict physical performance; and, in severe cases, reduced thoracic compliance, diminished vital capacity, and impaired cardiopulmonary function may occur [5].

Beyond the physical effects, AIS also entails profound psychological and psychosocial challenges during puberty, a particularly sensitive developmental period for the consolidation of self‐concept and personal values [6]. During adolescence, multiple stressors converge to intensify patients' psychological burden. On the one hand, they must constantly navigate the tension between adhering to brace treatment and avoiding negative attention or rejection from peers. On the other hand, they are prone to catastrophically anticipating and evaluating potential surgical risks and postoperative pain, while simultaneously experiencing inadequate family support, such as insufficient emotional reassurance, limited communication, and restricted involvement in decision‐making. Compared with adults, adolescents are still undergoing maturation in brain development, emotional regulation, and patterns of social attachment, rendering them more susceptible to pronounced mood fluctuations, excessive worry, and profound uncertainty about their future education, careers, and intimate relationships [7].

Family systems theory conceptualizes the family as an interdependent, dynamically regulated organic whole, emphasizing that members' emotions, behaviours, and roles do not exist in isolation but form mutually influencing feedback loops through interaction [8]. AIS patients are in a critical period of adolescent psychological development; the disease trajectory involves long‐term orthopaedic correction, rehabilitation, and appearance changes, impacting not only patients' self‐identity and emotions but also imposing a dual burden of caregiving and psychological support on caregivers. Given their young age and lack of disease management and self‐care abilities, patients rely on caregivers as their primary supporters. However, most caregivers face deficits in knowledge and caregiving skills, alongside psychological and financial burdens, throughout the diagnosis and caregiving process [9, 10]. Disease uncertainty—through information gaps, expectation discrepancies, and value conflicts—not only impairs patients' quality of life but also heightens caregiver stress. Prolonged caregiving predisposes caregivers to exhaustion and irritability; restricted social activities and reduced personal time can affect physical and mental state alterations, manifesting as varying degrees of anxiety and depression. The prevalence of anxiety and depression among parents of AIS patients is significantly higher than that of the general parental population [11]. Across the entire care continuum—from hospitalization to discharge and home‐based management—caregivers shoulder critical responsibilities, including medical communication, treatment decision‐making, daily care, and emotional support, while concurrently confronting potential health deterioration, emotional exhaustion, and social isolation. These multifaceted burdens not only undermine caregivers' own physical and mental health but also, through feedback loops within the family system, adversely affect patients' treatment adherence, psychological adjustment ability, and long‐term clinical and psychosocial outcomes. Therefore, from the perspective of family systems theory, identifying caregivers' unmet needs and enhancing their self‐efficacy and sense of control—thereby alleviating intrinsic psychological distress and strengthening their capacity and readiness to manage the patient's health status and medical demands—is crucial for optimizing the entire care continuum.

Despite the growing attention to the dyadic interaction between patients and caregivers, the existing evidence base primarily derives from fragmented, single qualitative studies. The diversity of these studies in methodological orientation and cultural context reflects both the exploratory and context‐dependent nature of qualitative inquiry and underscores the unique value of systematic aggregation and comparison through meta‐synthesis. A previous systematic review and meta‐synthesis [12] mainly focused on patients' lived experiences and has yet to systematically incorporate the caregiver perspective within a qualitative evidence synthesis framework. Given adolescents' high dependence on caregivers, caregivers' pivotal roles in treatment decision‐making and value orientation, and their potential moderating effect on patients' psychological outcomes, it is imperative to conduct a systematic review and meta‐synthesis that encompasses both patients and caregivers, thereby more comprehensively illuminating the dynamic processes and interaction mechanisms within the patient–caregiver dyad.

In summary, the present study conducted a systematic review and meta‐synthesis, within a qualitative evidence synthesis framework, of the subjective experiences and psychological burden of adolescents with AIS and their primary caregivers. The aim was to identify shared needs and key influencing factors, thereby providing practical implications and theoretical foundations for developing psychologically informed support strategies and continuity‐of‐care models tailored to the AIS population.

## 2 Materials and Methods

### 2.1 Study Design

This study initially focused on the psychological experiences of AIS patients. However, clinical practice has repeatedly revealed significant emotional interplay between caregivers and patients, and the existing literature contains only one qualitative synthesis exclusively from the patient perspective [12], suggesting the limited explanatory power of a single viewpoint. Based on these clinical observations and the current literature, this study also incorporated the caregiver perspective to more comprehensively elucidate the holistic impact of the disease on the family system and the interaction mechanisms between both parties.

This systematic review and qualitative meta‐analysis were based on the qualitative evidence synthesis (QES) framework and employed the meta‐aggregation method developed by the Joanna Briggs Institute (JBI) [13]. The QES framework is a universal methodological system for qualitative evidence synthesis, standardizing quality assessment, evidence integration, and evidence grading in qualitative research, and is compatible with various qualitative synthesis approaches. JBI meta‐aggregation is a standardized operational method within the QES framework, with its core being the faithful aggregation of original qualitative findings. Grounded in pragmatism, this method integrates findings from multiple qualitative studies to distil complex phenomena and is therefore appropriate for the present study. Data collation and interpretation followed established guidance for qualitative evidence synthesis, and reporting adhered strictly to the PRISMA statement to enhance transparency [14].

### 2.2 Search Strategy

We systematically searched five major databases: PubMed, Embase, CINAHL, PsycINFO, and Web of Science. The search covered the period from the inception of each database to April 2026. To ensure a comprehensive retrieval of relevant literature, we employed a combination of keyword searching and subject heading searching, using Boolean and proximity operators to link various search terms. Specifically, the search strategy comprised two concept groups: (1) population‐related terms (e.g., ‘adolescent idiopathic scoliosis’, ‘spinal deformity’, ‘scoliosis’) and (2) methodology‐related terms (e.g., ‘qualitative research’, ‘phenomenology’, ‘grounded theory’, ‘ethnography’, ‘narrative research’, ‘focus group’, ‘interview’, ‘thematic analysis’, ‘content analysis’). Within each concept group, terms were combined with ‘OR’, and the two groups were linked with ‘AND’. Taking PubMed as an example, the search strategy was: (‘Scoliosis’[Mesh] OR ‘Adolescent Idiopathic Scoliosis’[Title/Abstract] OR ‘Spinal Deformities’[Title/Abstract] OR ‘Progressive Scoliosis’[Title/Abstract]) AND (‘Qualitative Research’[Mesh] OR ‘Qualitative’[Title/Abstract] OR ‘Phenomenology’[Title/Abstract] OR ‘Grounded Theory’[Title/Abstract] OR ‘Ethnography’[Title/Abstract] OR ‘Narrative’[Title/Abstract] OR ‘Focus Group’[Title/Abstract] OR ‘Interview’[Title/Abstract] OR ‘Thematic Analysis’[Title/Abstract] OR ‘Content Analysis’ [Title/Abstract]). Additionally, we conducted a manual screening of the reference lists of included studies to supplement the database search for potentially missed relevant literature. No restrictions were imposed on publication type, year, or language throughout the search process to ensure broadness and inclusivity.

### 2.3 Inclusion and Exclusion Criteria

Eligibility criteria were developed using the PICoS framework recommended by the Joanna Briggs Institute (JBI) [15]:Inclusion criteria: (Ⅰ) Population (P): adolescents aged 10–18 years (inclusive) with a confirmed diagnosis of AIS and their caregivers; (Ⅱ) Phenomenon of interest (I): psychological experiences of AIS patients and caregivers; (Ⅲ) Context (Co): the full trajectory of AIS diagnosis and treatment (conservative observation, bracing, and surgery); (Ⅳ) Study design (S): qualitative studies employing methodologies such as phenomenology, grounded theory, and ethnography. Exclusion criteria: (Ⅰ) duplicate publications or incomplete data; (Ⅱ) inability to obtain full text; (Ⅲ) non‐English publications; (Ⅳ) mixed‐methods studies in which qualitative data could not be separately extracted.

### 2.4 Study Selection

Five researchers with expertise in evidence synthesis participated in the screening process. Cheng LY and Zhao JY independently screened the titles and abstracts simultaneously. The selection of full texts was completed by the other two authors, Hu X and Wang S, independently. During the title, abstract, and full‐text screening process, Qiu C also conducted an independent screening and review, serving as an arbitrator when differences of opinion arose. To ensure the consistency of judgement criteria between the two groups of screeners, Cheng LY, Zhao JY, Hu X, Wang S and Qiu C received dedicated training on the inclusion criteria before commencing full‐text screening, and calibration was performed using 30 studies that did not meet the inclusion criteria. The inter‐screener agreement between the two groups was calculated, with all results exceeding 90%. Furthermore, during the full‐text screening process, if Hu X and Wang S had doubts regarding a study's inclusion, it was necessary to review the judgment records of Cheng LY and Zhao JY from the title and abstract screening stage. Articles marked as ‘likely to be included’ by Cheng LY or Zhao JY at the title/abstract phase but subsequently excluded by Hu X or Wang S at the full‐text phase were automatically referred to Qiu C for final adjudication. Cross‐checks were performed throughout the process to ensure consistency. The screening process and results adhered to PRISMA guidelines [14].

### 2.5 Quality Appraisal

To ensure our findings are based on sufficient data and to avoid unreliable conclusions. The quality of the included studies and the effectiveness of the methods were evaluated independently by Hu X and Wang S using the CASP checklist [16]. This checklist lists 10 criteria aimed at determining the validity of the study results, the content of the results, and the applicability of the results. All review comments were resolved after discussion with a third reviewer.

### 2.6 Data Extraction and Synthesis

Hu X and Wang S independently read the full texts and extracted data, capturing all concepts presented across different contexts and study designs. Contextual information was simultaneously extracted, and study characteristics were summarized to facilitate assessment of the transferability of the synthesis. Data analysis and synthesis were conducted using thematic analysis in NVivo (Release 1.2). The specific process was as follows: first, line‐by‐line coding of the Results sections of the included studies was performed, generating open codes closely aligned with the original expressions; subsequently, through comparison of similarities and conceptual associations between codes, these were grouped into axial codes; axial codes were further aggregated into sub‐themes based on core foci, and ultimately integrated into the themes and conceptual framework presented in the results. The research team iteratively verified themes through repeated discussion and cross‐checking to ensure the consistency and trustworthiness of the analysis.

### 2.7 Assessment of Confidence in the Findings

We employed the Confidence in the Evidence from Reviews of Qualitative research (CERQual) approach [17] to evaluate the confidence in the synthesized findings. This tool assesses the quality of meta‐analysis findings across four dimensions: (1) methodological limitations; (2) relevance; (3) coherence of findings; and (4) adequacy of data. We adopted a tripartite arbitration mechanism involving two independent reviewers to assess the credibility of the meta‐analysis results, thereby determining the degree of concordance between the meta‐analysis conclusions and the actual situation of the research question.

## 3 Results

### 3.1 Study Selection

**Figure 1:**
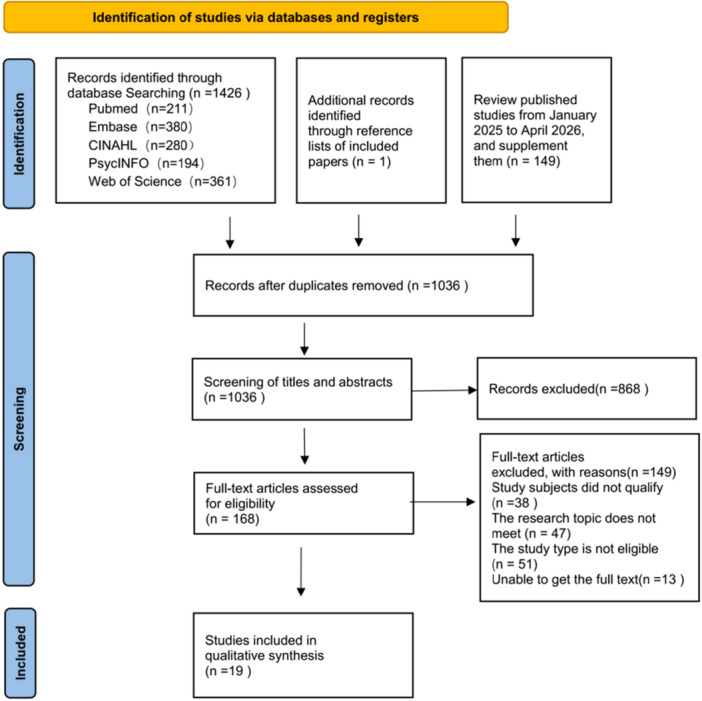
Inclusion in the literature screening process.

Using Zotero [18], a total of 520 duplicate records were identified and excluded. Two reviewers independently screened the titles and abstracts of the remaining 1036 records. Ultimately, 868 records were excluded based on the review topic and article type. Subsequently, the full texts of the remaining 168 articles were independently assessed by the same reviewers according to the inclusion criteria, and 19 studies were finally included (Figure 1). During the screening process, the reviewers maintained an inter‐rater agreement of at least 95%, and any disagreements were resolved through discussion with the third author.

**Figure 2:**
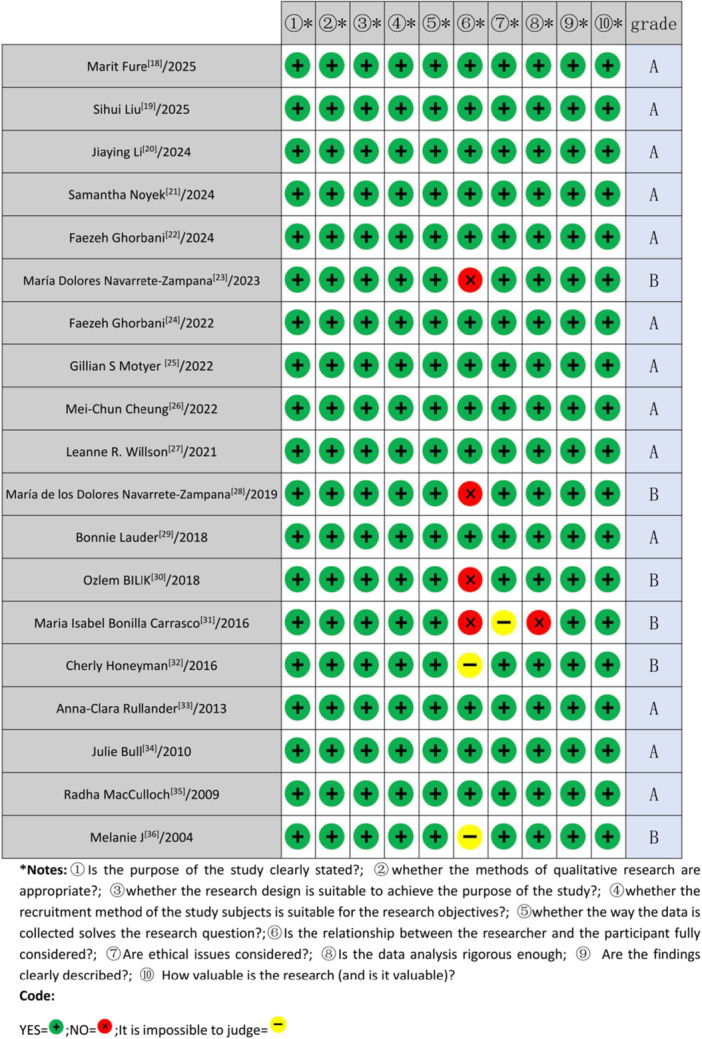
Critical appraisal skills programme, CASP.

All 19 studies were judged to have acceptable methodological quality (Figure 2).

A standardized data extraction form was developed based on the JBI Qualitative Assessment and Review Instrument (JBI‐QARI) checklist (see Tables 1 and 2).

**Table 1:** Basic characteristics of included qualitative studies.

Author/date of publication	Country	Phenomenon of interest	Subject investi	Age	Gender	Circumstances	Research method	Data collection
Marit Fure[19]/2025	America	Explores the bodily experiences of adolescents with AIS while receiving brace treatment.	Patients *n* = 13	12–15 years old	Women *n* = 10; Men *n* = 3	Seven wore a full‐time brace, three had transitioned from a full‐time brace to a nighttime brace and three only wore a nighttime brace	Exploratory qualitative research from the perspective of phenomenology	Semi‐structured interviews
Sihui Liu[20]/2025	China	To explore the obstacles and promoters faced by family caregivers of AIS patients in economic support agent decision‐making.	Patients *n* = 18	36–55 years old	Women *n* = 11; Men *n* = 7	Caregivers undergoing spinal surgery mainly during summer and winter vacations	Qualitative descriptive Application of COM‐B model	Semi‐structured interviews
Jiaying Li [21] /2024	Hong Kong, China	Adolescent idiopathic scoliosis: stressors, coping strategies, and mental health	Patients *n* = 12	10–18 years old	Women n = 7; men *n* = 5	8 were using braces and the remaining 4 were receiving physiotherapy	Descriptive qualitative research	Semi‐structured interviews
Samantha Noyek[22]/2024	Canada	Explore young people's experiences before, during, and after spinal fusion surgery to showcase their experiences beyond pain and pain.	Patients *n* = 20	12–18 years old	femininity *n* = 19; the male sex *n* = 1	Patients who have undergone spinal fusion surgery	Longitudinal observational study The social constructivist approach	Questionnaires, daily diaries, memory interviews, focus groups
Faezeh Ghorbani [23] /2024	America	To understand the brace adherence process for adolescent spinal deformities.	Patients *n* = 32; caregivers n = 32	Patients were 10–14 years of age	The patient's gender was not specified Caregivers (women *n* = 27; male *n* = 5)	Seventy‐four participants were inter viewed, including 32 adolescents treated with braces and their parents (27 mothers, five fathers), six orthotists, two physiotherapists, and two spine surgeons	Qualitative descriptive design, explanatory framework	Face‐to‐face interviews, in‐depth interviews, semi‐structured interviews
María Dolores Navarrete‐Zampana [24] /2023	Spain	Learn about the process of correction of limb deformity and postoperative transition in adolescent idiopathic scoliosis	Patients *n* = 22	12–18 years old	—	Has undergone corrective surgery	Symbolic interactionist approach	Semi‐structured interviews
Mei‐Chun Cheung[25]/2022	Hong Kong	To explore the subjective experience of patients during stent treatment, to understand the barriers they exist, and to make recommendations	patients *n* = 15	10–16 years old	Women *n* = 13; men *n* = 2	Patients currently receiving brace therapy	Qualitative methods	Semi‐structured in‐depth interviews
Gillian S Motyer[26]/2022	Ireland	A more comprehensive and in‐depth exploration of the psychosocial experience of patients prior to surgery	Patients *n* = 14	12–17 years	Women *n* = 10; men *n* = 4	Two participants had a confirmed surgery date. The remainder were waiting to receive a date or anticipated that they would be scheduled for surgery in future.	Qualitative methods	Semi‐structured interviews
Faezeh Ghorbani[27]/2022	USA	USALearn about the experiences of teenagers undergoing brace therapy during their school years, focusing on special children and their hidden problems.	Patients *n* = 32; caregivers *n* = 32	Patients were 10–14 years of age	The patient's gender was not specified Caregivers (27 women; 5 men)	Adolescents and Parents Receiving Orthopedic Treatment with Braces	Qualitative descriptive methods, explanatory frameworks	Semi‐structured, face‐to‐face, in‐depth interviews, telephone conversations
Leanne R. Willson[28]/2021	Canada	Determine what the needs of parents of children with scoliosis are and how they can be met at different levels of care	Caregiver *n* = 16	—	Women *n* = 12; men n = 4	Caregivers at different stages of treatment	Qualitative description	Semi‐structured interviews
María de los Dolores Navarrete‐Zampana[29]/2019	Spain	Learn about the pain experience after surgical correction of adolescent idiopathic scoliosis.	Patients *n* = 7	12–21 years old	Women *n* = 7	3–5 days after AIS corrective surgery	Interpreting the phenomenological approach	Semi‐structured interviews
Ozlem BILIK[30]/2018	Turkey	Uncover the short‐term experiences of adolescents and their families after scoliosis surgery	Patients *n* = 17; caregiver *n* = 9	Patients aged 12–18 years; age of caregiver not mentioned	—	Patients who have undergone spinal surgery	Qualitatively describe the design	In‐depth interviews
Bonnie Lauder[31]/2018	Australia	Explore and describe experiences of caring for children throughout the course of illness and treatment	Caregiver *n *= 12	The average age of caregivers is 39 years.	—	—	Qualitatively describe the design	Semi‐structured interviews
Cherly Honeyman[32]/2016	UK	Explore adolescents’ understanding of the perioperative experience and explore how to improve their experience	Patients *n* = 6	15–18 years old	Women *n* = 6	Patients who have undergone spinal fusion surgery	Phenomenological analysis, interpretivist paradigms	Focus groups, semi‐structured interviews
Maria Isabel Bonilla Carrasco[33]/2016	Spain	Explain the experience of being diagnosed with a physical deformity of idiopathic adolescent scoliosis	Patients *n* = 12	15–18 years old	Women *n* = 12	Patients who have undergone surgical treatment	Qualitative, phenomenology, hermeneutics	Semi‐structured interviews
Anna‐Clara Rullander[34]/2013	Sweden	Describe the narrative experience of an adolescent going through scoliosis surgery	Patients *n* = 6	8–18 years old	Women *n* = 4; men *n* = 2	Surgery on the spine	Qualitative descriptive methods	Open and semi‐structured interviews
Julie Bull[35]/2010	UK	Learn about the experiences of parents of children with scoliosis in diagnosis, surgical correction, and rehabilitation	Caregiver *n* = 12	—	Women *n* = 11; male *n* = 1	Caregivers of patients undergoing spinal surgery	Interpretive phenomenological analysis	Interviews and questionnaires
Radha MacCulloch[36]/2009	Canada	Identify the health‐specific needs of patients who have undergone or are about to undergo spine surgery for online information and support	Patients *n* = 11	10–18 years old	Women *n* = 9; men *n* = 2	Surgery performed or planned within 12 months	Describe qualitative research	Focus group method
Melanie J[37]/2004	America	From the perspective of current patients and parents, we will explore the issues related to the treatment of AIS (surgery and brace).	Patients *n* = 12; caregivers n = 10	Parental age is mentioned; the patient is 12–18 years of age	Gender of patients not specified; gender of caregivers (female *n* = 8; male *n* = 2)	Patients with AIS and their caregivers	Qualitative methods	Focus group method and interview method

**Table 2:** Key findings of included qualitative studies.

Date of publication	Number of topics	Topic name	Number of subtopics	Subtopic name
2025 [19]	3	(1) inhibited body; (2) alienated body; (3) disciplined body	—	—
2025 [20]	3	(1) Capability; (2) motivation; (3) opportunity	11	(1) Knowledge and skills; (2) physical and mental health; (3) alleviating the financial burden; (4) altruism and advocacy; (5) trust in financial assistance; (6) lack of confidence in application outcomes; (7) concerns about patients’ educational opportunities and privacy protection dilemma; (8) child's rebellion and non‐cooperation; (9) strict application conditions and complicated procedures; (10) family support; (11) social support;
2024 [21]	5	(1) disease‐ and treatment‐induced changes and stressors; (2) cognitive assessment and personal perceptions; (3) behavioural and emotional coping strategies(4) social interactions and social support; (5) deteriorating or thriving in psychological development and well being	17	(1) Physical activity restriction and body image changes; (2) burden and demand from bracing; (3) pain and time‐ consuming from non‐bracing treatments; (4) feeling different from others; (5) normalizing and looking at the bright side; (6) general acceptance after psychological adjustment; (7) adaptive coping and engagement in treatments; (8) emotional expression and modulation; (9) maladaptive behavioural coping and emotional suppression; (10) changes in social interactions and relationships; (11) excessive attention and psychological control; (12) sufficient instrumental support; (13) insufficient professional psychological support; (14) psychological distress and emotional reactions; (15) low self‐esteem and depressive symptoms; (16) development of a peaceful mindset and psychological resilience; (17) different spinal curve progression outcomes
2024 [22]	5	(1) body aesthetic versus machine; (2) expectations and anticipation of surgery and outcomes; (3) desire of normalcy and freedom; (4) navigating a hoped‐for positive surgery experience; and (5) the journey sculpts identity formation and sense of self.	—	—
2024 [23]	4	(1) Accepting the diagnosis; (2) orthotist interaction; (3) treatment acceptance; (4) living with a brace	13	(1) There is a problem;seeking for a cause; (2) finding the diagnosis; (3) referring to an orthotist; (4) first visit with the orthotic; (5) orthotists’ communication; (6) casting trauma(7);brace wearing; (8) brace bitter moon; (9) coping with activities of daily life; (10) social life adaptation; (11)admission/coping; (12) inner feelings; (13) coiance
2023 [24]	3	(1) The nature of the transition; (2) transition conditions; (3) response patterns	—	—
2022 [25]	3	(1) Physical discomfort due to brace material and design; (2) reluctance caused by the brace's visual appearance; (3) passive patient participation during the treatment process	—	—
2022 [26]	4	(1) Proceeding with caution; (2) am i different?; (3) an emotional journey; (4) no pain, no gain	4	(1) Appearance changes; (2) a hidden condition; (3) emotional rollercoaster; (4) not the only one
2022 [27]	3	(1) Concerns; (2) actual problems; (3) received supports	6	(1) Draw attention; (2) feeling different; (3) annoying; (4) reactions;physical limitations; (5) helpful; (6) empathy
2021 [28]	5	(1) Needing reliable medical information; (2) desiring information on complementary treatments; (3) wanting help in supporting and advocating for their child; (4) needing to protect the child and family; (5) seeking	—	—
2019 [29]	4	(1) influencing factors; (2) values and ideas; (3) coping mechanisms; (4) areas that could be improved	5	(1) The individual; (2) relationships/support; (3) the context; (4) attitudes to the process; (5) actions to mitigate pain
2018 [30]	5	(1) Physical complaints; (2) unfamiliar environment (operating room and intensive care unit) (3) emotional changes; (4) wanting parents’ accompaniment; (5) worry about future	7	(1) Pain; (2) vomiting, constipation and feeling hungry; (3) fear of death; (4) positive body image; (5) feeling good; (6) fear of reoperation; (7) lack of knowledge about life at home
2018 [31]	4	(1) Emotional rollercoaster ride; (2) lack of resources; (3) money talks; (4) pervasive burden	9	(1) I'm just an annoying mother; (2) self‐blame; (3) a depressive state; (4) just Google it; (5) there is no support; (6) public vs. private; (7) financial grief; (8) tyranny of distance; (9) it's physically exhausting
2016 [32]	4	(1) Shock, fears and worries; (2) parental interaction; (3) coping; (4) motivation and positivity	18	(1) Shock at diagnosis; (2) shock at decision of surgery; (3) fear of the unknown; (4) lack of control; (5) specific worries; (6) closeness; (7) advocacy; (8) support; (9) protection (10) normalization‐sleep; (11) food; (12) environment; (13) education; (14) conscious and sub‐conscious distraction; (15) active avoidance; (16) insignificance(17)self‐motivation‐actual and desired; (18) positive messages for others
2016 [33]	4	(1) Perception of spinal deformity; (2) characteristic symptoms of deformity; (3) impact of social relationships; (4) treatment methods and strategies	—	—
2013 [34]	3	(1) Emotional aspects; (2) physical aspects; (3) social aspects	14	(1) Fear; (2) nightmares; (3) nervousness; (4) mobilization; (5) scars; (6) different hip levels; (7) pain; (8) nausea; (9) urinary catheter; (10) appetite; (11) friends; (12) power; (13) coaching and comfort; (14) helplessness sports
2010 [35]	5	(1) Information: ‘a lot of unanswered questions’; (2) parenting role: ‘i felt i were on me own’ (3) confidence in health professionals: ‘pretty much every patient for himself’; (4) pain; (5) effect on life: ‘a hell of a roller coaster of worry and mixed emotions’	—	—
2009 [36]	9	(1) recovery at home; (2) recovery in hospital; (3) post‐surgical appearance; (4) emotional impact of surgery and coping; (5) intrusion of surgery and recovery of daily activities; (6) impact of surgery on school, peer relationships and other social interactions; (7) decisionmaking about surgery; (8) being in the operating room; (9) future worries	—	—

### 3.2 Assessment of Confidence in the Findings

Our appraisal indicated that the confidence levels for the seven themes ranged from moderate to high. Details are presented in Table 3.

**Table 3:** Evaluation table based on the results of Confidence in the Evidence from Reviews of Qualitative research.

Consolidate the results	Related research	①*	②*	③*	④*	CERQual outcome
Initial Diagnostic Phase: Multidimensional Responses to Acute Shock	14 items [19, 21, 22, 23, 24, 25, 26, 27, 30, 32, 33, 34, 35, 36]	A	A	A	A	Senior
Treatment Phase: Dynamic Adaptation Amid Ongoing Challenges	18 items [19, 21, 22, 23, 24, 25, 26, 27, 28, 29, 30, 31, 32, 33, 34, 35, 36, 37]	A	A	A	A	Senior
Recovery Phase: Divergent Pathways of Growth and Residual Trauma	11 items [19, 20, 21, 22, 23, 24, 29, 32, 33, 34, 36]	A	A	A	A	Senior
Structural burden	8 items [20, 21, 22, 23, 28, 30, 31, 35]	A	A	A	A	Intermediate
Imbalance in Support Systems	13 items [20, 21, 22, 23, 26, 28, 30, 31, 32, 33, 34, 36, 37]	A	A	A	B	Senior
Emotional Exhaustion and Psychological Consequences	9 items [20, 21, 22, 23, 28, 30, 31, 35, 36]	A	A	A	B	Intermediate
conflictual intimacy	9 items [20, 21, 22, 25, 28, 30, 34, 35, 36]	A	A	A	A	Intermediate

*Note:* Code: No downgrades = A; downgrade by 1 level = B.

* ① Methodological limitations; ② Correlation; ③ Consistency of results; ④ Data sufficiency.

### 3.3 Thematic Synthesis

**Figure 3:**
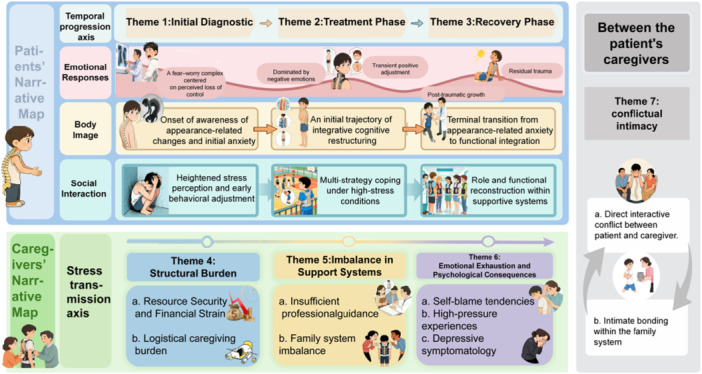
Summary of the thematic synthesis findings. The figure was created based on information obtained from Doubao and publicly available online sources.

This review synthesized the psychosocial experiences of both adolescents with AIS and their caregivers into two complementary illness narrative maps (Figure 3). The first map follows the temporal trajectory of the illness, illustrating patients' psychological adaptation from the initial shock of diagnosis to subsequent reconstruction across three interrelated dimensions: emotional responses, body image, and social interactions. The second map is organized according to levels of caregiving burden, depicting the progressive accumulation of caregiver stress arising from resource depletion, inadequate support, and psychological exhaustion. These two trajectories intersect within the family system, together portraying a comprehensive psychosocial landscape of the AIS experience.

#### 3.3.1 Psychosocial Burden From the Perspective of AIS Patients

##### 3.3.1.1 Theme 1: Initial Diagnostic Phase: Multidimensional Responses to Acute Shock [19, 21, 22, 23, 24, 25, 26, 27, 29, 32, 33, 34, 35, 36]

###### 3.3.1.1.1 Emotional Responses

A fear–worry complex centred on perceived loss of control. This integrated synthesis found that negative emotions in patients with AIS are not fragmented psychological distress, but rather a composite response pattern driven by a sense of loss of control and compounded by fear and worry. The illness deprives adolescents of bodily autonomy and developmental predictability; the triple uncontrollability of curve progression, treatment pathways, and long‐term prognosis constitutes the core source of loss of control. At initial diagnosis, this loss of control erupts as shock and denial *(*‘That can't be my spine, is that my spine? It must be somebody else's spine’ [25]); as disease awareness deepens, it transforms into persistent fear, including instinctive resistance to the visible constraint of the brace and amplification of perceived risks of surgery and complications (‘was quite fearful…they tell me that they have to operate, to open my back and insert a prosthesis…that hit me quite hard’ [23]), often accompanied by sleep disturbances. On this basis, fear further generalizes into diffuse worry about negative peer evaluation at school, brace‐related shame, and surgical recurrence, while long‐term anxiety about growth trajectory and functional prognosis magnifies subjective loss of control.

###### 3.3.1.1.2 Body Image

Onset of awareness of appearance‐related changes and initial anxiety. The research indicates that the core of body image distress in AIS patients is the awakening of awareness of altered appearance, namely a sudden shift from unawareness of bodily symmetry to hypervigilance regarding deformity, triggering anxiety. Adolescence is a sensitive period for body image formation; trunk asymmetry directly challenges patients‘ core perception of a normal body, rapidly binding appearance to self‐worth and triggering identity conflict and decreased self‐confidence (‘I feel like my shoulders are uneven…you can tell at a glance’ [33]). Patients view themselves from an external observer's perspective, marking asymmetry as abnormal and internalizing societal body standards, with concerns progressively developing from dissatisfaction to shame and low self‐esteem (‘I wouldn't be able to live with it… because I just wouldn't be able to like my body’ [25]). Their distress arises more from the anxiety of anticipated scrutiny than from actual social rejection (‘I asked a few friends, and some said they never realized it’ [27]).

###### 3.3.1.1.3 Social Interaction

Heightened stress perception and early behavioural adjustment. The study found that changes in social interaction during early diagnosis are essentially anticipatory perception of potential stress and preventive behavioural adjustments. Even before experiencing explicit social rejection, patients withdraw from social engagement proactively due to body image awakening and anticipated negative evaluation, seeking psychological safety (‘I'm a girl who keeps to herself and swallows problems… […] I have always been like this… I have never expressed my feelings…’ [24]). School is a primary stress setting: rehabilitation therapy reduces study time and academic participation, physical limitations decrease peer activity engagement, together reinforcing role marginalization (‘Physiotherapy does [reduce my time]… I had less time to study because of physiotherapy’ [19]). Patients manage body visibility through strategies such as loose clothing and avoiding exposure‐prone activities to control others' perceptions and maintain minimal social safety (‘I cannot visit a swimming pool or go to a beach because I don't want anyone to see the ugly scar, and I don't want anyone to touch it or ask about it’ [33]). These behaviours, though seemingly passive, are situational protective strategies developed in the absence of mature coping skills, aimed at managing others' perceptions through visibility control, thereby preserving a minimal sense of social safety.

##### 3.3.1.2 Theme 2: Treatment Phase: Dynamic Adaptation Amid Ongoing Challenges [19, 21, 22, 23, 24, 25, 26, 27, 28, 29, 30, 31, 32, 33, 34, 35, 36, 37]

###### 3.3.1.2.1 Emotional Responses

This integrated synthesis found that during treatment, patients‘ emotions are characterised by predominant negative emotions, with positive adjustments being brief and fragile. The uncertainty of non‐surgical treatment efficacy constitutes a persistent stressor; physical exposure during examinations and the continuous visibility of the brace repeatedly activate shame and embarrassment (‘We conducted body scans of the adolescents who had to expose their trunks entirely. However, they often felt embarrassed during the process’ [22]); mechanical restriction of the brace, skin damage, and postoperative acute pain add psychological burden through physical discomfort, leading to transient reduction in emotion regulation and irritability. Catastrophic pain experiences during and after surgery trigger existential threat perceptions (‘I thought I was going to die. The pain was very severe. It was compared to a disaster, a feeling that one was going to die’ [33]). Against the backdrop of prolonged treatment and outcome uncertainty, patients become hypervigilant to potential worsening of the condition during daily activities, prompting highly avoidant and cautious behavioural patterns. However, within the same treatment phase, transient positive adjustment triggered by cognitive reappraisal can also be observed: some patients reframe orthotic intervention as a routine physiological aid, thereby reconstructing the meaning of treatment and temporarily alleviating stigma, while also holding anticipatory positive emotions centred on aesthetic improvement after surgical correction (‘This was the day I got my grad dress back from alterations. I remember genuinely feeling pretty and confident with myself for the first time in a long time’ [21]). Nevertheless, such positive activation is highly dependent on smooth treatment progress and is vulnerable to disruption by technical setbacks such as orthosis malfunction, exhibiting marked context dependence.

###### 3.3.1.2.2 Body Image

An initial trajectory of integrative cognitive restructuring. The synthesis reveals that during treatment, body image disturbance exhibits a progressive trajectory of adaptation from rejection towards initial cognitive integration. Visible somatic markers left by surgery (‘attempted to walk straight and avoided lazing on beaches or pools, because the defect is evident in the eyes of others’ [32]) inscribe the pain of disease onto the body's surface through visual impact, while the persistent somatosensory anomaly induced by brace‐wearing intensifies the alienating experience of the body as a foreign object. Together, these constitute a dual assault on the integrity of the body schema. The anticipated gaze and labelling evaluations from the social environment further drive patients to adopt systematic stigma management strategies, manifested as visual information control mediated by clothing and even compensatory behaviours that sacrifice physical comfort to maintain the integrity of their social image (‘I wear a sweater to school even in summer to hide the brace since my school uniform is in white, my classmates will see it’ [29]). While these behaviours mitigate social anxiety in the short term, they also reinforce the intrinsic belief that bodily abnormality must be concealed. Concurrently, the preliminary initiation of a cognitive reframing pathway can be observed during the treatment period, manifesting as attempts to accept disease‐related bodily changes. Through cognitive reappraisal mechanisms, some patients gradually dissolve the foreign body sensation associated with the brace, achieving a perceptual re‐integration from an external orthopaedic device to an extension of the body (‘When I first got my brace, it felt separate from me, but now I see it as a part of my body’ [22]). Such processes of re‐signification lower the symbolic meaning of the brace as a catalyst for stigma by altering the cognitive representation of the orthopaedic intervention, laying a psychological foundation for deeper body acceptance later on. Moreover, outcome expectancies oriented towards long‐term postural improvement and functional recovery constitute the core motivation underpinning treatment adherence (‘I have confidence in this brace… Precisely for this reason, I am motivated to use the orthosis more frequently’ [26]), suggesting that a goal‐oriented cognitive framework can partially buffer the erosive effects of immediate physical discomfort and negative body image on treatment persistence. However, body acceptance at this stage remains partial, having not yet achieved a stable reconstruction of overall body identity.

###### 3.3.1.2.3 Social Interaction

Multi‐strategy coping under high‐stress conditions. Entering the treatment phase, social interaction pressures become fully manifest, with the core feature being the threat of multiple role losses. Academic impairment, reduced physical activity participation, and inflexible normative demands in institutional settings collectively constitute cumulative burdens on daily role performance, depriving patients of identities as students, athletes, and friends, and forcing abandonment of previous interests (‘I am always very unhappy… I used to love playing basketball, [now] I'm not happy at the thought of having to wear a brace after I've played basketball’ [34]). Under this pressure, patients‘ coping strategies show significant polarization, reflecting different psychological regulatory pathways. Maladaptive coping includes concealing brace adherence from medical staff, actively withdrawing from social settings, and suppressing emotional expression, ultimately exacerbating treatment resistance and social withdrawal (‘I put the brace on to go to school and take it off at school and put it in my locker until the end of the day. I just hated wearing it’ [28]), Adaptive coping strategies unfold along three dimensions: active help‐seeking, behavioural adjustment, and cognitive reappraisal. Patients expand coping resources by seeking informational and emotional support from professionals and peer networks (‘My friends say that I wear a brace now as if I am wearing armor’ [24]), they use concrete pain management to enhance situational control and proactively request reasonable accommodations from the environment to maintain role function; cognitive adaptive efforts are reflected in autonomous participation in treatment decisions, which restores a sense of control over disease management and provides a psychological foundation for rebuilding positive self‐identity.

##### 3.3.1.3 Theme 3: Recovery Phase: Divergent Pathways of Growth and Residual Trauma [19, 20, 21, 22, 23, 24, 29, 32, 33, 34, 36]

###### 3.3.1.3.1 Emotional Responses

Coexistence of post‐traumatic growth and residual trauma. Entering the later recovery phase, the emotional adaptation of AIS patients exhibits a heterogeneous pattern of divergence, characterized by the coexistence of posttraumatic growth and residual trauma reactions. The psychological trajectory of one cohort of patients embodies the typical features of posttraumatic growth, the core mechanism of which lies in transforming the illness experience from a painful memory into a narrative resource for personal growth through cognitive reappraisal and meaning‐making. By comparing pre‐ and post‐treatment postural improvements, patients re‐affirm self‐worth and decision‐making courage (‘I felt like I succeeded. I felt so proud of myself, and this journey had given me, so much…’ [21]), and on this basis, develop an integrated life narrative characterized by acceptance and equanimity. At the level of social cognition, developmental gains are also evident, manifested as an enhanced capacity to decode others' social signals subtly, distinguishing inquisitive inquiries from derogatory judgments and adopting more adaptive communication strategies accordingly. A more pronounced marker of growth is reflected in the proactive reshaping of identity narratives; patients transition from passive recipients of therapeutic interventions to active agents who empower others with their lived experience, achieving an identity leap from patient to victor through public sharing (‘I actually started the account [personal scoliosis account]… share my story… we all were called the scoliosis warriors…’ [21]), and in the process, distill survival beliefs centred on resilience and hope, ultimately forging an adaptive outcome with a sense of mastery and self‐efficacy at its psychological core. However, the psychological landscape of another cohort of patients remains persistently encumbered by unresolved traumatic imprints, manifesting as intrusive symptoms and hyperarousal responses consistent with the post‐traumatic stress disorder spectrum. Fear memories associated with the risk of intraoperative nerve injury repeatedly intrude into consciousness in the form of nightmares. Even after the conclusion of treatment protocols, functional recovery in some patients lags behind expectations, with ongoing high dependency on caregivers in basic daily activities, reflecting difficulty in exiting the sick role and impaired autonomy restoration. Furthermore, the persistent pain reported by some patients and its resistance to conventional analgesia (‘I… […] have had a hard time because of the pain […] and the pain during the previous year… it was too much… no matter how much medicine I too’ [32]) suggests that chronic pain and psychological distress may form a mutually reinforcing vicious cycle, indicating that residual somatic and psychological trauma constitutes a non‐negligible, persistent burden during the later recovery phase.

###### 3.3.1.3.2 Body Image

Terminal transition from appearance‐related anxiety to functional integration. In the later recovery phase, patients' body image cognition shifts from concern with appearance to acceptance of function and experience. Some patients reinterpret surgical scars as marks of growth and courage rather than stigma (‘The Mark of Courage’ [34]). Humanistic care from healthcare professionals and social comparison with wardmates facilitate body acceptance and reduce loneliness (‘when you're there you kind of feel like there's other people and there's always someone worse than you and you feel… it's going to be okay like I'm not the only one’ [22]). Accepting statements from family members dissolve shame about bodily differences (‘Mom said ‘your back is just different from others, not bad‐looking’’ [20]), while positive feedback from peers corrects previous concerns about others' evaluations and repairs relationships strained by the illness. These factors collectively enable some patients to actively participate in creating disease‐related educational content, regain discursive agency over their own experiences, and ultimately incorporate illness‐related bodily changes into their life narratives, achieving functional integration.

###### 3.3.1.3.3 Social Interaction

Role and functional reconstruction within supportive systems. During the recovery period, the focus of social interaction shifts towards the gradual restoration of role function and continuity in daily life within supportive relationship networks. The experience of returning to everyday contexts alongside peers helps to repair the sense of normalcy that was disrupted during the treatment phase, providing a contextual anchor for a smooth transition of identity (‘going out of the house to spend time with peers as methods of coping during the post‐surgical recovery period’ [33]). Peer groups, through their sustained presence and emotional investment, provide non‐obligatory social connections; such bonds play a unique function in mending the social isolation caused by the disease and rebuilding basic trust in interpersonal accessibility. Some patients further reconstruct meaning in everyday life by proactively expanding the boundaries of their interests and abilities; the renewed sense of competence catalyzed by objective improvements in physical function not only fortifies self‐efficacy but also re‐establishes individual value through aesthetic and expressive activities. In this process, peer and family systems form a mutually complementary external support architecture, significantly promoting psychological recovery and the generation of positive meaning. The function of the family system at this stage manifests as a holistic mobilization oriented towards crisis adaptation; family members, by adjusting role boundaries and interaction patterns, jointly shoulder the emotional labour and instrumental tasks of disease management, thereby maintaining the stability and resilience of the family system (‘parents, as their main carers, had a positive attitude to their recovery, acting in a way that supported them during the same’ [23]). Parental accompaniment not only constitutes the material basis for emotional security but also serves as a buffer and advocate when confronting external medical and social environments. By constructing the family as a predictable and reliable safe harbour, parents enable patients to complete the functional transition from enduring social pressure to adopting proactive coping and adaptation.

The psychological adaptation process of AIS patients and their caregivers is not linear but rather a dynamic trajectory of recurrent oscillation between loss of control and reconstruction. This process can be roughly divided into three phases. At initial diagnosis, the disease acts as an acute stressor that rapidly undermines adolescents' sense of control over their own bodies and future development. During the prolonged treatment phase, persistent fear and physical pain form the daily context, compelling both parties to continuously seek adaptive strategies while enduring distress. In the later recovery phase, some patients are able to transform their illness experience into personal growth resources and achieve identity reconstruction, whereas others remain trapped in traumatic experiences and struggle to regain psychological equilibrium. Ultimately, the key determinant of prognostic differences is not the Cobb angle or the treatment modality per se, but rather whether the patient can rebuild bodily autonomy and complete the transition from identity confusion to self‐identity confirmation.

#### 3.3.2 Psychosocial Burden of AIS Caregivers

##### 3.3.2.1 Theme 4: Structural Burden: Long‐Term Financial Strain, Time, and Logistical Demands [20, 21, 22, 23, 28, 30, 31, 35]

###### 3.3.2.1.1 Resource‐Safeguarding Pressures

This type of strain focuses on the lack of material capital and institutional support required for fulfilling the caregiving role, with core manifestations including depletion of economic resources and absence of social security. Research reveals that role conflict between caregiving responsibilities and paid labour is the primary driver of family economic vulnerability; some caregivers are forced to exit the labour market owing to the indivisibility and intensive time demands of care (‘… I actually ended up resigning from work and became a full time carer’ [30]), leading to structural disruption of household income. Direct medical expenditures combined with indirect costs of cross‐regional care‐seeking significantly raise financial pressure. Gaps in medical insurance coverage and limited commercial insurance reimbursement force some families to finance treatment through high‐cost borrowing (‘…… Having to borrow money at high interest rates’ [22]). Economic constraints thus permeate downward to medical decision‐making, distorting treatment choices from clinical need toward affordability. Resource strain also manifests in functional failure of social support networks: institutional‐level systemic safety nets for families with special‐needs children are lacking; community‐level weak screening and health literacy systems lead to inadequate social awareness and lack of substantive service linkage (‘I don't think the government or anyone understands the cost involved with a child with a special condition, they have no idea’ [20]); emotional responses in informal networks remain at the level of pity without translating into actionable resource support, resulting in a substantial attenuation of support effectiveness (‘…… then you get quite a lot of pity rather than support, and there is a difference between pity and support you know’ [35]). These triple ruptures in economic, institutional, and community resources together constitute the core of caregivers‘ resource strain.

###### 3.3.2.1.2 Logistical Caregiving Burden

The study found that caregivers' temporal autonomy is significantly co‐opted by the demands of scoliosis treatment and rehabilitation, with the rhythm of life rigidly tethered to medical procedures. The inherent uncertainty of the disease process, such as repeated and unpredictable delays in surgical scheduling, directly dismantles caregivers' capacity for future planning and their sense of control over temporal rhythms, leaving family life in a state of suspension. This uncertainty, intertwined with the intense density of caregiving tasks, triggers a dual disruption of temporal and spatial order. At the macro level, the planning space for long‐term living arrangements and leisure activities is severely compressed (‘I think the worst thing is that you can't plan your holidays’ [35])*;* at the micro level, personal time resources are compressed to the limit, with caregivers required to continuously invest in immediate physical care labour such as patient repositioning and pain episode management, leading to severe sleep deprivation and energy depletion (‘Lifting her, moving her, sleep deprivation, when she is in pain I am up… there are some nights where you are lucky to get to sleep in two or three days’ [30]). Logistical pressures further spill over into the domain of caregivers' social role fulfilment, manifested as a forced reduction in work participation and a marginalized contraction of social networks. The geographical dimension also constitutes a significant source of logistical pressure; for families residing in rural or remote areas, the long journey to and from medical centres not only consumes time and physical energy but also transforms into a persistent psychological burden of worry due to the patient's discomfort during transport (‘…always difficult, the drive home is scary, he is in pain when we are driving home, so I always worry…’ [22]). These multidimensional daily overloads mutually reinforce each other, rendering caregiving labour the core variable dominating the rhythm of family life and leading to a flattening of the family's functional structure.

##### 3.3.2.2 Theme 5 Imbalance in Support Systems: Dual Lack of Professional Information and Emotional Support [20, 21, 22, 23, 26, 28, 30, 31, 32, 33, 34, 36, 37]

###### 3.3.2.2.1 Insufficient Professional Guidance

The synthesis reveals that information provided by healthcare institutions falls far short of caregivers‘ cognitive needs; most caregivers acquire knowledge through fragmented self‐learning via the internet, facing risks of information distortion and misinformation (‘I was reading, watching YouTube, I taught myself about it, because they didn't give me anything’ [30]); the field lacks mature patient peer‐support organizations, creating a structural gap in experiential support. A more serious problem is the skills gap in caregiving practice: at critical transition points in medical care, such as transfer from intensive care to general ward, caregivers experience intense helplessness and feelings of abandonment due to lack of necessary operational guidance and psychological preparation (‘but when we got on the ward, I felt I were on me own. The were just… [] I were just frightened, you know, in case she relapsed or whatever’ [34]); during the transition from inpatient treatment to home rehabilitation, the systematic absence of care management guidance directly leads to trial‐and‐error costs and even preventable secondary health damage (‘……but suffice it to say that largely owing to my own ignorance, the two ‘gaping' areas became infected with Staph Aureus (not MRSA), and we spent weeks tending them under the care of the GP’ [34]). These information gaps and skills deficits interweave, leaving caregivers facing challenges exceeding their coping capacity at every stage of disease management.

###### 3.3.2.2.2 Family System Imbalance

This category of adversity focuses on the systematic impact of caregiving burden on the internal relational structure and daily operational patterns of the family, essentially representing the transmission and diffusion of disease stress from the individual patient to the family system. At the level of the marital subsystem, the high intensity and protracted nature of caregiving tasks lead both spouses to concentrate their limited psychological resources on the child's needs, resulting in a significant reduction in investment in maintaining the couple's relationship. Consequently, the intimacy and communication quality of the marital relationship deteriorate, and the emotional bond tends to loosen (‘……It's been a long time since we sat down to talk’ [26]). Persistent physical and mental exhaustion and emotional tension further erode the family's emotional climate, placing caregivers in a state of heightened irritability, with a resultant increase in the frequency of conflict and negative emotional expression within family interactions. Notably, caregivers' own emotional needs are often relegated to a position of neglect within the family; when family attention is intensely focused on the child's disease progression, the fatigue and vulnerability of the caregiver as an independent individual struggle to garner sufficient attention and response, thereby breeding a profound experience of loneliness (‘The family focused solely on the child's condition, with no one inquiring about my fatigue’ [23]). At the level of daily family order, the frequency of family members’ participation in routine leisure and social activities significantly diminishes. The rhythm of family life is completely co‐opted by medical appointments and caregiving demands, with social networks contracting accordingly, and the family system demonstrating a tendency towards functional unidimensionality. Unequal access to information also constitutes a potential trigger for estrangement in couple relationships; when one party is deeply involved in medical communication while the other remains on the informational periphery, the consequent discrepancy in understanding the disease process can exacerbate a sense of detachment between them (‘… My wife was given [information from the clinic it] but I didn't really pay attention to that’ [28]). Against this backdrop, establishing connections with caregivers who share similar experiences becomes an important outlet for mitigating feelings of isolation, as they can offer emotional validation and substantive assistance based on a common experiential ground.

##### 3.3.2.3 Theme 6 Emotional Exhaustion and Psychological Consequences: From Self‐Blame and High Pressure to Risk of Depressive Symptomatology [20, 21, 22, 23, 28, 30, 31, 35, 36]

###### 3.3.2.3.1 Self‐Blame Tendencies

The study found that during the initial onset and diagnosis phase, caregivers commonly experience intense self‐accountability for failing to recognize symptoms early, interpreting the missed window for early intervention as a failure of their personal caregiving duty. This attribution style overlooks the objective reality that the onset of AIS is often insidious and early physical signs are difficult to detect (‘How could I not have noticed earlier?… As her parents, I was powerless to change this’ [30]). Some caregivers even extend the attribution of the disease to non‐directly associated factors such as behaviours during pregnancy, reflecting a tendency for individuals, in the absence of accurate aetiological information, to fill cognitive gaps through retrospective attribution. When caregivers learn of a genetic link to the disease, self‐blame escalates to its peak; the explicitness of the genetic chain shifts the locus of responsibility from caregiving negligence to inherent transmission, deepening the orientation of guilt from the behavioural level to the level of life transmission itself. Upon entering the treatment phase, the visible suffering inflicted on the child by invasive procedures directly triggers caregivers' mirror empathy, plunging them into remorse and ambivalence over treatment decisions (‘We were unaware that the surgery posed such significant difficulties for him, to the extent that he was unwilling to undergo any intervention’ [35]). Meanwhile, the disclosure of extreme complications, such as the risk of paralysis, within surgical informed consent forms superimposes a profound fear of potential future irreversible harm upon the existing foundation of self‐blame, causing decision‐making pressure and moral burden to escalate simultaneously. Furthermore, objective constraints in accessing financial resources and allocating caregiving attention further reinforce caregivers' negative self‐evaluation of their role fulfilment. In extreme cases, persistently accumulated self‐blame can evolve into a total negation of self‐worth and a wavering of existential meaning (‘…at that stage I was suicidal…I had a sick son, I blamed myself’ [23]).

###### 3.3.2.3.2 High‐Pressure Experiences

The synthesis reveals that this category of adversity focuses on the persistent state of pressure that caregivers endure throughout the entire disease trajectory, building upon the foundation of self‐blame. Its core stressor is not a single event, but a chronic anticipation of uncertainty regarding treatment outcomes and caregiving efficacy. At the long‐term level, caregivers' concerns about the child's future quality of life and the long‐term impact of the disease constitute background anxiety; this sense of suspension regarding an unknowable future keeps caregivers in a state of continuous psychological alert (‘How is he going to be 10 years from now, 20 years from now? How is this going to affect his quality of life?’ [30]). During the observation and bracing treatment phase, caregivers must seek a balance between fulfilling the child's emotional needs and strictly enforcing treatment regimens. The process of supervising adherence is often accompanied by parent‐child conflict and emotional drain, trapping caregivers in a state of role tension. Concurrently, the passive waiting for disease progression and the sense of uncontrollability over adherence efficacy further intensify caregivers' feelings of powerlessness and anxiety. In the perioperative period, the focus of caregivers' pressure shifts to scrutiny and concern regarding the reliability of the medical care system; when the child is outside the scope of family supervision, external dependence on care quality coupled with an absence of oversight forms a new source of anxiety (‘I was constantly worried that the nurses/auxiliaries were not attending to her as regularly as I had expected…’ [36]). During the home rehabilitation phase, even though the main treatment phase has concluded, caregivers continue to face fears of disease recurrence and doubts about whether their own caregiving skills are sufficient to support recovery needs, indicating that the psychological impact of uncertainty does not naturally abate with the end of treatment (‘…… I were just frightened, you know, in case she relapsed or whatever’ [28]).

###### 3.3.2.3.3 Depressive Symptomatology

This category of adversity reveals the deeper psychological consequences resulting from the compounding of long‐term self‐blame and high‐pressure states, the essence of which is the systemic depletion of caregivers' physical and mental resources and a comprehensive collapse of their sense of self‐worth. At the somatic level, chronic caregiving stress directly leads to persistent energy drain and physical fatigue. The high intensity and ceaseless nature of caregiving labour keep caregivers in a perpetual state of energy deficit, while the sleep deprivation induced by the 24‐h continuous care demands during the postoperative period further undermines the physiological foundation for physical recovery and emotional regulation (‘24‐hour postoperative care for children with sleep deprivation of less than 4 hours’ [22]). Physical exhaustion and sleep insufficiency jointly constitute the somatic preconditions for depressive manifestations such as low mood. At the emotional and relational level, when the child reacts with resistance due to the painful experiences of treatment and projects negative feelings onto the caregiver, the caregiver not only endures the role pressure of enforcing pain but also faces impaired parent‐child relationships and an identity crisis—transforming from protector into an object of the child's aversion. This reversal of the relationship dynamic profoundly shakes the caregiver's foundation of self‐worth (‘…… I was now all of a sudden seen and perceived as an annoying mother’ [35]). Concurrently, an irreconcilable internal conflict arises between the caregiver's high empathy for the child's suffering and the role requirement of persisting with treatment, trapping them in an emotional laceration of being heartbroken yet compelled to be resolute. When treatment outcomes fall short of expectations, and long‐accumulated hopes repeatedly encounter setbacks in reality, caregivers' psychological resilience is progressively depleted, their capacity for positive future anticipation markedly diminishes, and feelings of dashed hope and powerlessness mutually reinforce each other, forming an inescapable negative cognitive cycle. Ultimately, the persistent superimposition of the aforementioned multiple factors can lead to changes at the personality level and a holistic decline in the caregiver's vital energy. In extreme cases, suicidal tendencies emerge, signifying that the depressive mood has attained a severity that endangers life safety (‘So at that stage I gave up, at that stage I was suicidal, I didn't want to live anymore’ [23]).

Based on the above integrative analysis from the caregiver's perspective, a cascading mechanism of ‘resource rupture, coping incapacitation, and psychological collapse’ emerges among structural burden, systemic support deficiency, and emotional exhaustion. Financial vulnerability and logistical overload collectively constitute the basal pressure of the caregiving context, depriving the family of the material buffer and temporal elasticity needed to cope with the disease. Upon this soil of resource depletion, the absence of professional guidance and the relational attrition within the family system further dismantle the caregiver's coping capacity and emotional replenishment, plunging them into a dual isolation where no one instructs them what to do and no one shares the burden they bear. When external support retreats comprehensively while caregiving demands continue to mount, self‐blame and high pressure, as psychological variables, are continuously activated, ultimately exhausting the caregiver's physical and mental reserves and steering them towards a depressive outcome characterized by somatic depletion, emotional laceration, and a wavering of existential meaning.

#### 3.3.3 Between the Patient's Caregivers

##### 3.3.3.1 Theme 7: Conflictual Intimacy [20, 21, 22, 25, 28, 30, 34, 35, 36]

Family systems theory posits that the family is an emotional unit composed of interconnected members, and individual behaviour and emotions cannot be understood in isolation from interaction patterns within the system. Disease, as an external stressor, simultaneously impacts internal hierarchical boundaries and emotional bonds within the family, giving rise to two distinct but interwoven forms of conflict, which we term conflictual intimacy.

###### 3.3.3.1.1 Direct Interactive Conflict Between Patient and Caregiver

Research reveals that long‐term caregiving alters parent–child interaction patterns functionally: attention to treatment adherence extends to daily life details, compressing adolescents‘ autonomy in decision‐making; individual preferences are overridden by medical considerations, provoking resistance against control (‘It [Uneven shoulders] will not affect my choice of clothing. But if the clothes I choose can't hide the uneven shoulders, my mother will say you have even shoulders, choose another one… So I, I don't have the freedom to choose clothes’ [20]). Conflicts around brace wearing are most typical: caregivers adopt monitoring and coercion to ensure treatment effectiveness, while adolescents express resistance covertly or overtly, forming a cycle of monitoring, avoidance, coercion, and confrontation. Younger children, highly dependent on parents, tend to adopt strategies of overt compliance and covert resistance, maintaining surface balance through non‐public non‐cooperation (‘……My mom says you are going to have to come upstairs and show me that you are wearing it to bed…….I would go up, show her, go down……I sleep in the basement so nobody knows. So, I am like, not wearing it’ [36]); direct coercion often triggers open conflict with emotional involvement, turning caregiving situations into battlefields of emotional consumption (‘…There were some days we fought over it. There were tears going’ [35]). In postoperative pain contexts, children, with limited emotion regulation, transform physical discomfort into crying and pressure on caregivers, further intensifying interactional tension and caregivers' implementation dilemmas. Such conflicts essentially originate from deep mutual concern and represent a tension‐laden expression of intimacy under disease pressure.

###### 3.3.3.1.2 Intimate Bonding Within the Family System

From the perspective of family systems theory, the AIS disease and treatment process directly challenge the caregiver's core family role as protector. When the child faces the risk of somatic trauma and the parent is powerless to directly intervene or eliminate the suffering, the sense of control and efficacy presupposed by the protector identity is fundamentally called into question. This experience of obstructed role execution constitutes a significant source of deep‐seated anxiety for caregivers. To compensate for this sense of role defeat, some caregivers develop behavioural tendencies of hypervigilance and intensified protection; although they may recognize that their reactions might exceed objective necessity, they find it difficult to escape a state of persistent monitoring for potential risks. This relationship pattern, characterized by overprotection, is summarized in family interactions as a pervasive state (‘We got… basically this [sense of] over protective mother syndrome’ [35]). The caregiver's high empathy for the suffering endured by the child leads them to map the child's painful experience as proof of their own unfulfilled protective duty, further reinforcing the internal moral pressure. However, it is precisely within this process of role being challenged that another dimension of intimate relationships—the deepening of emotional bonds—is also quietly occurring. Older adolescents gradually develop, over the course of caregiving, an awareness and understanding of the caregiver's dedication, capable of interpreting unconditional acceptance and love from the parent's attention to bodily details. This cognitive shift allows the relationship to transition from a unidirectional model of management and compliance towards a bidirectional model of understanding and empathy (‘I still remember how my mother felt my spine before the operation. She said my body was perfect in her eyes’ [28]). Concurrently, caregivers may also, under specific circumstances, receive emotional feedback from their children, discovering that the child's latent need for their presence far exceeds expectations. Such discoveries shatter the caregiver's pre‐existing judgements about the closeness or distance of the parent‐child relationship (‘The intensive care unit nurse called me. My daughter wanted to see me very much…….I got surprised when I received that call’ [35]), forging a more intimate emotional bond.

It therefore becomes evident that the disease simultaneously undermines caregivers' confidence in their protective role while creating opportunities to reaffirm and strengthen parent–child relationships. Conflict and intimacy are not opposing constructs but coexist throughout the family's process of adaptation. Protective anxiety and adolescent resistance, role disruption and emotional reconnection, interact continuously, together constituting the distinctive relational tension experienced by families living with AIS.

## 4 Discussion

a. Summary of key findings

This study provides an integrative analysis of the psychosocial burden experienced by adolescents with AIS and their caregivers across the entire disease trajectory, revealing key findings at three levels.

At the patient level, the psychological trajectory demonstrates a distinct phase‐specific pattern, evolving from an initial loss of control and heightened body image awareness at diagnosis, through cognitive adaptation and diverse coping strategies dominated by negative affect during treatment, to divergent outcomes characterised by both post‐traumatic growth and persistent psychological distress during recovery. Shame related to physical appearance and self‐objectification persist throughout the disease trajectory, representing the central psychological burden experienced by patients.

At the caregiver level, psychological burden follows a clear cascading pathway from structural stress to psychological exhaustion. Financial vulnerability and logistical burden constitute the underlying sources of stress, while inadequate professional guidance and deterioration in family relationships progressively erode caregivers' coping resources. Ultimately, the cumulative effects of self‐blame and sustained psychological pressure contribute to depressive symptomatology.

At the dyadic level, a distinctive relational pattern characterised by role conflict and emotional tension emerges between patients and caregivers. This relationship reflects an ongoing tension between caregivers' protective instincts and adolescents’ pursuit of autonomy, in which conflict and emotional closeness coexist dynamically within the family system.

b. Systematic comparison with existing literature

Compared with the existing literature, this meta‐synthesis deepens the understanding of the specificity of the psychosocial burden of AIS at the following levels. First, regarding the nature of psychological distress, previous studies have confirmed that AIS can lead to long‐term impairment of psychosocial function, associated with depression, insomnia, and somatic symptoms [38], with Sanders et al. [39] reporting clinically significant distress in 32% of patients. This study found that the core driver of such distress is not the life‐threatening nature of the disease itself, but rather social evaluative anxiety and self‐identity conflict triggered by visible appearance changes. In contrast to diseases like leukemia [40] or cancer [41], where the threat is directly life‐oriented, the anxiety of AIS patients is concentrated on themes related to appearance normalcy, such as bodily symmetry, clothing concealment, and peer acceptance. The underlying reason for this difference is that AIS predominantly occurs during adolescence, a developmental stage of heightened sensitivity to self‐identity, body image, and peer relationships, where physical appearance should ideally be approaching its ideal form, but instead deviates from expectations due to spinal deformity. Hair loss or scars resulting from cancer treatment, while also triggering body image distress, are largely interpreted by patients as badges of their fight against the disease; moreover, such changes typically occur in adulthood, exerting a different qualitative impact on core self‐identity. AIS patients, lacking a devastating life‐threatening outcome to counterbalance their appearance anxiety, struggle to garner societal sympathy and instead endure persistent worry about their appearance being scrutinized. Second, regarding the maintenance mechanism of body image disturbance, AIS brace treatment typically requires wearing for 23 h a day over several years, and the brace may still be detectable under clothing. This prolonged, non‐concealable disease label leads to chronic stigma and social isolation. Chemotherapy‐induced alopecia in leukemia is temporary, and post‐surgical cancer scars can be covered by clothing; however, AIS patients bear the daily psychological burden stemming from the treatment tool itself rather than the disease's consequences, a phenomenon relatively rare in chronic illness. Arslani et al. [42] noted widespread self‐esteem impairment due to appearance; this study supplements the mechanism by elucidating its pathway: perceived disease stigma activates a self‐objectifying perspective, which in turn triggers preventive avoidance in social interactions, forming a negative cycle of appearance concern, anticipated anxiety, and social withdrawal. Regarding the phased nature of psychological evolution, AIS stands in stark contrast to adolescent chronic conditions like Type 1 diabetes [43] that require long‐term daily management. The psychological burden in diabetic patients revolves around blood sugar monitoring, dietary control, and complication prevention, lacking distinct temporal nodes and drastic fluctuations in treatment intensity. The treatment trajectory of AIS, however, encompasses phase transitions with significantly differing intensities—diagnostic confirmation, brace‐wearing decisions, surgical timing decisions, immediate postoperative recovery, and long‐term rehabilitation—each node constituting a concentrated shock to the patient's psychological state. Particularly unique is the fact that treatment decisions in AIS often evolve into a structural tension between patients and caregivers: patients resist bracing or surgery due to appearance shame and a need for autonomy, while caregivers mandate compliance due to fear of disease progression. This direct interactive conflict, originating from the clash between adolescent autonomy needs and the sense of being controlled by treatment, rarely appears with such high frequency and intensity in other chronic conditions, further amplifying the emotional load and coping difficulty for patients across treatment stages. Finally, the study found that age is a key variable influencing the psychological experience of AIS patients. Younger patients are more concerned with treatment‐induced physical discomfort and life restrictions, whereas older patients, with gradually established self‐identity and heightened social needs, are more sensitive to appearance anomalies and social evaluation, with a tighter coupling between self‐perceived burden and self‐worth. This is consistent with the finding by Gallant [43] that age is negatively correlated with psychological status in AIS patients. These differences collectively indicate that the psychosocial burden of AIS patients is not a generic manifestation of psychological responses to chronic illness, but a disease‐specific pattern jointly shaped by its adolescent onset, the visibility and protracted nature of treatment, and the unique autonomy‐versus‐control tension inherent in the therapeutic relationship.

Compared with caregivers of severe diseases such as those requiring hemodialysis [44] or cancer [45], the psychosocial burden of AIS caregivers exhibits both commonalities—manifested as role overload, financial and time pressures, and emotional exhaustion—and specific differences in manifestations and sources of stress. The burden of hemodialysis caregivers is concentrated on the persistent encroachment of regularly scheduled treatments upon the family's daily rhythm. The core stress for cancer caregivers revolves around the difficult trade‐off between survival prognosis and treatment side effects. In contrast, the decisional pressure for AIS caregivers is more prominent; the phased transitions in treatment pathways, the fragmented distribution of professional information, and the potential divergence in treatment outcome expectations between medical teams and families [46] place caregivers in a prolonged cognitive tug‐of‐war between risk aversion and aesthetic prognosis. Furthermore, while caregivers of other chronic conditions also face structural burdens of declining income and insufficient social support [47, 48], AIS, due to its treatment cycle spanning the entire adolescent growth period and its rehabilitation requirements involving long‐term commitment to posture management and functional training, presents a more pronounced persistence in temporal and logistical pressures. This study found that caregivers' psychological burden peaks during the patient's surgical period, specifically manifesting as panic over losing control of the medical process during surgery, fear of their own failure as protectors postoperatively, and a continuous tug‐of‐war between hope and risk throughout. These characteristics have not been systematically described in prior caregiver research.

c. Interpretation of unexpected or novel findings

This study is the first to systematically identify a unique relational pattern between AIS patients and their caregivers, characterized by both intimacy and conflict, which can be termed ‘conflictual intimacy’. Family systems theory [49] helps explain its underlying mechanisms. During adolescence, a critical developmental stage characterized by increasing autonomy and individuation, the long‐term care dependency imposed by the disease forces both parties into a conflict between emotional fusion and individuation needs. Patients desire independence like their peers, yet cannot extricate themselves from dependence on parents for bracing, surgical care, follow‐up accompaniment, and decision support; caregivers, driven by fear of disease progression, tend towards excessive involvement and protection, gradually transitioning from emotional supporters to medical managers. When the caregiver's controlling behaviours continuously collide with the patient's assertiveness, a push‐pull dynamic emerges where closeness triggers argument and distance breeds worry. Unlike cancer or other adult‐dominated illnesses, the trajectory of AIS spans the entirety of adolescence; this prolonged uncertainty prevents the family from temporarily suspending autonomy needs as they might when facing an acute crisis, causing conflictual intimacy to persist and potentially evolve into a stable family interaction pattern. More notably, in some families, the conflict can spill over to third parties, for instance, when the caregiver allies with the physician to pressure the patient, or the patient counters the parents by refusing treatment—a phenomenon described as triangulation in family systems theory. The above mechanisms reveal that conflictual intimacy is not simply poor family relations but an inevitable product of the interplay between disease characteristics, developmental stage, and family dynamics. This mutual interlocking of dependency and control is the driving force behind conflictual intimacy; both parties do not lack love for each other, but rather cannot find a balance point that allows for both closeness and respect for each other's boundaries. This finding transcends the limitation of prior research that discussed the psychological burdens of patients and caregivers separately, shifting the target of clinical intervention from the individual to the dyadic interaction, and providing a direct evidence base for boundary‐restructuring interventions grounded in family systems theory.

Furthermore, across the disease trajectory, the patient's three‐phase psychological evolution and the caregiver's accumulated burden mutually shape and cyclically reinforce each other. During the initial diagnosis phase, the patient's sense of loss of control and fear directly trigger the caregiver's role conflict and financial strain; if healthcare institutions can provide clear diagnostic pathways and psychological support at this stage, the caregiver's burden may be confined to information‐seeking rather than escalating to role collapse. During the treatment phase, the patient's psychological burden amplifies the caregiver's exhaustion through an emotional contagion, while the patient's transient positive adaptation can conversely offer glimmers of hope. The window for intervention at this phase lies in establishing an asymmetric division of labour: the caregiver assumes the core responsibility of medical decision‐making while deliberately preserving space for the patient's autonomous self‐management, thereby alleviating protector stress and delaying emotional exhaustion. In the later recovery phase, the caregiver's state of depletion determines whether the patient can achieve posttraumatic growth. If the caregiver is already severely depleted, the quality of their emotional support diminishes, making it difficult for the patient to complete the cognitive restructuring from appearance anxiety to functional integration. Conversely, the caregiver, acting as a secure base, can propel the patient's growth. The key intervention point at this phase is the gradual transfer of responsibility; the caregiver steps back from comprehensive management into a reserve support role, encouraging the patient to independently manage their health and social life. This simultaneously eases the caregiver's burden and bolsters the patient's sense of self‐efficacy. In summary, seizing the intervention points at each phase for targeted action can break the vicious cycle of burden accumulation and promote dyadic co‐adaptation.

d. Theoretical significance and implications for evidence‐based practice

At the theoretical level, a core contribution of this study lies in revealing that the psychosocial adaptation of AIS patients and their caregivers is not a static aggregation of various distresses, but a dynamic process. This dynamic process originates from the perturbation triggered by the disease's visibility, proceeds through individual cognitive appraisal, then through intra‐family role negotiation, ultimately diverging towards varied adaptive outcomes. Prior studies [50, 51] largely examined patients’ emotional responses, body image, and social interactions in isolation, failing to integrate them with the family system, thereby fragmenting the transmission pathways of disease stress between the individual and the system. This integrative analysis elucidates the temporal logic from a processual perspective: the disease first shocks self‐identity through the sudden visible change in physical appearance, activating emotional responses centred on a sense of loss of control and driving preventive social withdrawal. Upon entering treatment, bracing and surgical interventions shift bodily difference from concealable to persistently exposed, forcing both patient and family to synchronously enter a phase of intensive cognitive and behavioural adaptation. The divergent outcomes in the recovery phase hinge on whether the individual can integrate the illness experience into a coherent life narrative, and whether the family system can achieve a return from disease‐management mode to a normal life rhythm. More critically, this study found that the relationship between patient and caregiver is not a unidirectional pathway of support provision; rather, a bidirectional feedback loop forms within the structural tension between the caregiver's frustrated protector role and the adolescent's assertion of autonomy. Caregivers compensate for role inadequacy through intensified control, while patients respond to autonomy deprivation with avoidance or resistance; the emotions and behaviours of both parties mutually amplify each other within the interaction, collectively shaping the adaptation trajectory of the system. This theoretical integration organically bridges the boundary permeability concept from family systems theory with process models of stress and coping, not only filling the long‐standing gap in the AIS field regarding an integrative psychological mechanism framework but also pointing to a necessary methodological premise: studying patients and caregivers as separate units would preclude capturing the true sources of dynamism within disease adaptation.

Based on the integrative findings of this study, the following intervention directions are empirically substantiated. First, patient support should adhere to the principle of phase‐matched delivery. During the initial diagnosis phase, informational support aimed at restoring a sense of controllability should be prioritized; during treatment, cognitive‐behavioural interventions targeting body image [52], peer support groups [53], and mindfulness training [54] should be integrated into standard care; in the recovery phase, narrative therapy should be employed to facilitate posttraumatic growth [55]. Clinicians should assess body image disturbance at every medical visit. Second, caregiver support must simultaneously cover both instrumental and affective dimensions. The study reveals that caregivers' emotional exhaustion is rooted in information scarcity, skills deficits, and disruption of family roles; thus, phase‐specific information provision, caregiving skills training, and the establishment of intra‐family collaborative mechanisms are directly necessary [56]. Third, family‐unit communication interventions are more fundamental than separate interventions. The study identifies protective silence, medicalized control, and parent‐child conflict as core manifestations of interactive predicaments. Therefore, guiding caregivers to gradually share disease information commensurate with the child's age and enhancing adolescent autonomy through shared decision‐making are actionable pathways to reduce resistance behaviours [57]. The above directions stem directly from the chain of qualitative evidence in this study and can be regarded as the core content of evidence‐based implications. Additionally, based on this research, the authors propose the following extended recommendations: at the school level, spinal health knowledge should be incorporated into health education curricula, and teacher training on disease awareness should be provided to foster an inclusive campus environment [58]; at the community level, community health service centres should be utilized to integrate local medical resources and establish family support groups to shorten pathways to information access and emotional resonance; at the policy level, attention must be directed to the economic accessibility of screening, diagnosis, and treatment, alleviating family burdens through channels such as charitable funds; at the hospital level, multidisciplinary collaboration processes should be optimized, establishing a continuous information dissemination mechanism spanning from preoperative education to postoperative rehabilitation, and strengthening linkages with communities and schools. The qualitative synthesis results of this study directly support the identification and description of psychological burden themes, whereas the design of a multi‐level intervention system constitutes an authorial theoretical derivation, the effectiveness of which has not been directly validated in this study and is more suitable as a starting point for future interventional research.

## 5 Limitations

This study has several aspects that warrant continuous refinement. The search was conducted without restrictions on publication year or language, which, while broadening the inclusion scope, may have rendered the comparability of treatment experiences across different eras and the findings from non‐English‐speaking cultures less readily apparent due to the wide temporal span and the exclusive inclusion of English‐language literature. Some studies were excluded due to inability to obtain full texts or to independently extract qualitative data; although efforts were made to compensate through interlibrary loans and reference list backtracking, room for potential selective inclusion may remain. Mixed‐methods studies where the qualitative data could not be isolated were also excluded, leaving the coverage of evidence sources slightly less than complete. The screening process was completed by different researcher pairings in phases; although consistency exceeded 90% after unified training and calibration with 30 articles, and coherence was maintained by reviewing prior judgement records, the lack of a single, invariant team throughout the entire process may introduce judgement variability. Furthermore, the proportion of female patients in the included studies generally exceeded 70%, which aligns with the epidemiological characteristics of AIS, yet also implies that the conclusions predominantly reflect female experiences; extrapolation to male patients should be approached with caution. Future research should aim to include multilingual literature and incorporate more male samples or conduct gender‐comparative analyses to consolidate and broaden the robustness of these conclusions.

## 6 Conclusion

This meta‐synthesis reveals that the psychological adaptation of AIS patients undergoes a phased evolution from a sense of loss of control at diagnosis to adaptation during treatment, and finally to divergent outcomes in the recovery phase. Caregivers, meanwhile, endure a persistent structural burden centred on resource rupture and role frustration. The two parties are locked in a bidirectional feedback loop shaped by the tension between protection and autonomy. Clinical practice must take the family system as the intervention unit, addressing patients' needs in a phase‐specific manner while simultaneously alleviating caregivers' informational isolation and decision‐making pressure to interrupt the continuous reinforcement of negative feedback. Future research can, based on the bidirectional interaction framework synthesized in this analysis, develop and validate family‐system‐based intervention protocols, with a focus on evaluating the actual effects of improved information sharing and role negotiation on patients' adaptive outcomes and caregivers' emotional burden.

## 7 Relevance to Clinical Practice

This study builds on the perspective of patients with adolescent idiopathic scoliosis by further integrating the psychological dimension of caregivers, establishing an evidence‐based framework for psychological nursing that spans the entire disease process. This framework aligns with the concept of stage‐specific, level‐based, and population‐tailored psychological intervention, reflecting a people‐oriented and holistic humanistic care approach. It not only responds to the growing emphasis on psychosocial support in modern medicine but also contributes to enhancing the quality of healthcare services and improving the patient healthcare experience.

## Author Contributions

**Shuang Wang:** writing – original draft, writing – review and editing, methodology; data curation, investigation, conceptualization. **Jiayi Zhao:** project administration, conceptualization, software. **Xiong Hu:** methodology, formal analysis, supervision. **Yanli Yuan:** conceptualization, supervision, data curation. **Yingying Xu:** investigation, methodology, project administration. **Ting Kan:** conceptualization, writing – review and editing, validation. **Lingyan Cheng:** conceptualization, project administration, visualization. **Chen Qiu:** writing – review and editing, funding acquisition, methodology.

## Ethics Statement

Ethical approval was not required for this study as it is a synthesis of previously published research evidence.

## Conflicts of Interest

The authors declare no conflicts of interest.

## Supporting information


Supporting File

## Data Availability

The data that support the findings of this study are available from the corresponding author upon reasonable request. All data analysed in this systematic review are derived from the published qualitative studies listed in the reference list. The authors have archived the characteristic extraction tables, original theme, and participant quotation coding records (NVivo (Release 1.2) project export files) of all included studies. These data can be requested from the corresponding author upon reasonable request.
